# Impact of primary food allergies on the introduction of other foods amongst Canadian children

**DOI:** 10.1186/1710-1492-8-S1-A10

**Published:** 2012-11-02

**Authors:** Mary McHenry, Wade Watson

**Affiliations:** 1 Department of Pediatrics, Faculty of Medicine, Dalhousie University, Halifax NS, Canada; 2 Division of Allergy, IWK Health Centre, Halifax NS, Canada

## Background

Food-allergic children frequently avoid other foods. We hypothesized that parents of food-allergic children are not given consistent advice regarding introduction of allergenic foods; that these foods are avoided or delayed; and that there is significant anxiety when introducing new foods.

## Methods

An online survey was administered via *Anaphylaxis Canada*’s website to Canadian parents and caregivers who are registered members of this organization and who have a child with a food allergy.

## Results

644 parents completed the online survey (60% male children, average age at diagnosis 21.8 months). The most common allergies were peanut (49%), milk (23%), and egg (18%). 51% of families were given advice regarding the introduction of other allergenic foods, 97% followed through with this advice. 72% were told to avoid certain foods, 41% to delay certain foods, and 14% were given varied advice. 58% of parents avoided or delayed other highly allergenic foods, mainly due to a fear of allergic reaction or anaphylaxis (93%). 69% of children did not have an allergic reaction when these foods were introduced. 68% of parents felt moderate or high levels of anxiety when introducing other foods.

## Conclusions

Families of children with food allergies receive varied advice regarding the introduction of new foods. The majority of children did not have an allergic reaction to the new food, even if it was initially avoided or delayed. Most parents feel moderate to high levels of anxiety when introducing new foods to their children. A more consistent approach to this advice may decrease parental anxiety.

